# The Matrix Receptor CD44 Is Present in Astrocytes throughout the Human Central Nervous System and Accumulates in Hypoxia and Seizures

**DOI:** 10.3390/cells13020129

**Published:** 2024-01-10

**Authors:** Osama Al-Dalahmah, Alexander A. Sosunov, Yu Sun, Yang Liu, Nacoya Madden, E. Sander Connolly, Carol M. Troy, Guy M. McKhann, James E. Goldman

**Affiliations:** 1 Department of Pathology and Cell Biology, Vagelos College of Physicians and Surgeons, Columbia University Irving Medical Center and the New York Presbyterian Hospital, New York, NY 10032, USA; 2 Department of Neurosurgery, Vagelos College of Physicians and Surgeons, Columbia University Irving Medical Center and the New York Presbyterian Hospital, New York, NY 10032, USAesc5@cumc.columbia.edu (E.S.C.);; 3 Department of Pathology, Albany Medical Center, Albany, NY 12208, USA; 4 Department of Neurology, Vagelos College of Physicians and Surgeons, Columbia University Irving Medical Center and the New York Presbyterian Hospital, New York, NY 10032, USA; 5 The Taub Institute, Columbia University Irving Medical Center, New York, NY 10032, USA

**Keywords:** CD44, astrocytes, hypoxia, seizures, interlaminar astrocytes

## Abstract

In the mammalian isocortex, CD44, a cell surface receptor for extracellular matrix molecules, is present in pial-based and fibrous astrocytes of white matter but not in protoplasmic astrocytes. In the hominid isocortex, CD44+ astrocytes comprise the subpial “interlaminar” astrocytes, sending long processes into the cortex. The hippocampus also contains similar astrocytes. We have examined all levels of the human central nervous system and found CD44+ astrocytes in every region. Astrocytes in white matter and astrocytes that interact with large blood vessels but not with capillaries in gray matter are CD44+, the latter extending long processes into the parenchyma. Motor neurons in the brainstem and spinal cord, such as oculomotor, facial, hypoglossal, and in the anterior horn of the spinal cord, are surrounded by CD44+ processes, contrasting with neurons in the cortex, basal ganglia, and thalamus. We found CD44+ processes that intercalate between ependymal cells to reach the ventricle. We also found CD44+ astrocytes in the molecular layer of the cerebellar cortex. Protoplasmic astrocytes, which do not normally contain CD44, acquire it in pathologies like hypoxia and seizures. The pervasive and inducible expression of CD44 in astrocytes is a novel finding that lays the foundations for functional studies into the significance of CD44 in health and disease.

## 1. Introduction

The interlaminar astrocytes of the mammalian isocortex were first described well over one hundred years ago [1] and further characterized in recent years [2,3,4]. These astrocytes display marked differences from the better-known, bushy, protoplasmic astrocytes of the cortex. They project multiple, long, unbranched cell processes, usually oriented orthogonally to the pial surface, through several cortical layers, forming cascades of parallel processes. A subset of astrocytes in the deep cortical layers near the subcortical white matter also projects similar, radially oriented processes into the deeper cortical layers. Immunocytochemical studies reveal that these long-process astrocytes contain CD44 [5,6], a cell surface receptor for extracellular matrix molecules, including osteopontin, hyaluronan, laminin, and integrins [7]. They are different from the protoplasmic astrocytes, which do not contain detectable levels of CD44 [6,8], not only in morphology but also by being low in glutamine synthetase and the two plasmalemmal glutamate transporters, EAAT1 and EAAT2 [6]. The astrocytes in white matter are also CD44+ [6,8,9,10,11]. However, CD44 can be increased in all types of astrocytes in response to pathological conditions [6,8].

We have now examined other parts of the human central nervous system (CNS) and found that CD44+ astrocytes are common in every part of the CNS, including cortices, striatum, thalamus, brainstem, cerebellum, and spinal cord. In all areas, we found CD44 associated with long-process astrocytes. CD44+ astrocytes populate white matter, subependymal zones, and subpial areas. We found that the proximity of CD44+ processes to neuronal cell bodies varies considerably. In particular, unlike neurons in the isocortex and diencephalon, the motor neurons of the brainstem and spinal cord show a close apposition of CD44+ processes and are even encircled by these processes. We raise the issue of whether this apposition may suggest a novel, intimate relationship between neurons and CD44+ astrocytes for specific neuronal classes.

In this study, we have also examined biopsy and autopsy material from individuals who had experienced hypoxic events and seizures and found that protoplasmic astrocytes accumulate CD44 in these pathological conditions. Furthermore, we have investigated rat models of ischemia and seizures, finding that in those brains, protoplasmic astrocytes also acquire CD44.

## 2. Materials and Methods

### 2.1. Human Autopsy and Biopsy Material

Eight autopsy brains, fixed in 10% formalin for 10 days after removal, were sectioned coronally and samples from all areas of the CNS, including superior frontal, cingulate, and striate cortices with subcortical white matter, hippocampus with temporal isocortex, basal ganglia, thalamus, midbrain, pons, medulla, cerebellum with cortex and dentate nucleus, and spinal cord were removed and embedded in paraffin blocks, cut at 7 μm thickness, and mounted on glass slides. Patients’ ages ranged from 27 to 74; 6 were female, and 2 were male. None of these individuals displayed neurological symptoms, and none of the brains showed evidence of neuropathology except for mild atherosclerosis and/or arteriolosclerosis with acute but mild hypoxic/ischemic changes. In addition, we sampled sections of anterior striatum from 3 more autopsies (ages in years and sex 1M (male), 76F (female), and 94F), sections of hippocampus from 8 more autopsies (ages in years and sex: 1M, 2M, 5M, 7M, 73M, 71M, 76M, and 66M), and sections of spinal cord from 4 more autopsies (ages in years and sex: 27F, 66M, 80M, and 80M). We also sampled sets of sections of cerebral hemispheres containing the subependymal zone at the lateral ventricle from 8 fetal and neonatal brains, ranging in age from 19 to 40 weeks of gestation and 1 day to 7 weeks of postnatal life. All autopsies were performed with the consent of the next of kin, and all protocols were approved by the Institutional Review Board of Columbia University Medical Center.

### 2.2. Epilepsy Surgical Specimens

We examined 9 samples of temporal isocortex of patients with mesial temporal sclerosis (ages ranged from 12 to 55; 6M and 3F). We also examined 5 samples of hippocampi from patients with mesial temporal sclerosis (ages ranged from 9 to 66; 3M and 2F) obtained from surgical resections from patients with medically intractable epilepsy. We did not include specimens from seizure patients with brain tumors, vascular malformations, cortical dysplasias, and inflammatory and infectious disorders, although we found that many of them also show CD44+ astrocytes.

### 2.3. Hypoxia Specimens

Eight neurosurgical specimens that represented hypoxic/ischemic insults were selected. We used both the time between the initial clinical presentation and the biopsy and the neuropathology to classify these as acute, subacute, or chronic hypoxic/ischemic changes, acute within a few days, and showing eosinophilic neuronal change but no reactivities of blood vessels, astrocytes, or microglia, subacute between 10 and 14 days and showing proliferative vasculature and reactive astrocytes and microglia with macrophages, and chronic more than 14 days and showing tissue necrosis with foamy macrophages. Some of the biopsies showed acute and subacute or subacute and chronic changes, indicating progressive changes in the evolution of the lesions. The age range was 19–82, 3M and 5F. CD44-positive astrocytes were counted in three 20X (objective numerical aperture 0.40) power fields from each case. Sample *t*-tests were performed between pairs of the three groups.

### 2.4. Rodent Hypoxia/Ischemia and Seizure Models

Adult male rats were housed in standard cages with free access to food and water on a 12 h light/dark cycle. All procedures performed on animals were approved by Columbia University’s Institutional Animal Care and Use Committee and conducted according to Institutional and Federal guidelines.

### 2.5. Stroke/Transient Middle Cerebral Artery Occlusion (tMCAO)

Wistar rats (275–300 g) were subjected to unilateral tMCAO using intraluminal vascular occlusion. Animals were anesthetized with halothane in a mix of 70% nitrous oxide/30% oxygen, and core temperatures were maintained at 37  °C throughout the entire procedure and for 60 min after reperfusion. The right common carotid artery, the right external carotid artery, and the right internal carotid artery were exposed and isolated. MCAO was accomplished by advancing a 25 mm 4–0 nylon suture with a blunted silicone tip (outer diameter, 0.38 mm) through an incision in the external carotid artery until the suture was 18 mm past the carotid bifurcation. MCAO was confirmed by transcranial measurements of cerebral blood flow via laser Doppler flowmetry (Periflux System 5000; Perimed, Inc., Järfälla, Sweden). After 120 min, the suture was removed, and reperfusion was confirmed by laser Doppler flowmetry. After 7 days, animals were deeply anesthetized with an overdose of ketamine/xylazine and perfused with 4% (*v*/*v*). After perfusion, brains were removed and additionally fixed in 4% PFA in phosphate-buffered saline (PBS) for 14–18 h (4 °C). Forty-micron sections were prepared with a vibratome (Leica VT1000S, Wetzlar, Germany) and stored in cryoprotectant solution at −20 °C. After perfusion, other brains were dissected and incubated at 4 °C with 4% (*w*/*v*) PFA (24 h), PBS (24 h), and 30% sucrose (*v*/*v*) in PBS, then embedded in O.C.T. compound and 20 µm cryosections were prepared.

### 2.6. Pilocarpine-Induced Status Epilepticus

Seizures were produced in rats with pilocarpine, as described elsewhere [12]. After premedication with scopolamine (5 mg/kg, i.p.) to prevent the effects of peripheral cholinergic stimulation, pilocarpine (330 mg/kg, i.p.) was administered to Sprague–Dawley rats (100–150 g) to induce seizures. Seizures were graded on the modified Racine scale [13], and only animals with grade 4–5 seizures for 2 h were used in experiments. After 2 h of continuous seizures, ketamine (80 mg/kg, i.p.) was administered to stop seizures, and a second dose (40 mg/kg, i.p.) was administered if seizures did not stop in 10 min after the first. Animals were deeply anesthetized 3 days post-seizures and perfused as above. After perfusion, brains were removed and processed as above.

### 2.7. Immunostaining and Antibodies

Formalin-fixed paraffin-embedded (FFPE) sections were stained using hematoxylin and eosin (H&E). Immunohistochemistry with positive and negative controls was performed using an antibody to CD44 (Roche, mouse monoclonal antibody pre-diluted at 1:100 and run on the Ventana platform using the Ultraview DAB kit). For immunofluorescence staining on tissue sections from the rat MCAO occlusions and pilocarpine-induced seizures, we used a CD44 mouse monoclonal antibody (1:500, OX49/ab238464, Abcam, Cambridge, UK) and a glial fibrillary acidic protein (GFAP) rabbit polyclonal (1:1000, Z 0334, Dako, Carpinteria, CA, USA). Secondary antibodies conjugated to fluorophores: goat anti-mouse Alexa Fluor^TM^ 488, 594, and 633, goat anti-rabbit Alexa Fluor^TM^ 488 and 594, and donkey anti-goat Alexa Fluor^TM^ 488, 594, and 633 (1:300, ThermoFisher Scientific, Eugene, OR, USA). After blocking with 10% (*v*/*v*) normal goat (or donkey) serum (30 min, RT = 20–22 °C), free-floating sections were incubated in a mixture of primary antibodies overnight (4 °C). Alexa Fluor^TM^-conjugated secondary antibodies were used for 1 h at RT = 20–22 °C). For visualization of nuclei, DAPI (5 μg/mL; D9542, Sigma-Aldrich, St. Louis, MI, USA) was applied with the secondary antibodies. Blocking serum, primary, and secondary antibodies were applied in 0.2% Triton X-100 in PBS. Sections for fluorescence microscopy were mounted on slides in Vectashield (Vector Laboratories, Burlingame, CA, USA). To control for the specificity of immunostaining, primary antibodies were omitted and substituted with appropriate normal serum. Slides were viewed using a confocal microscope (Nikon Ti Eclipse, Tokyo, Japan). For immunofluorescence staining of cerebellar sections, we used 5–7 µm thick FFPE sections, which were immunostained on a Leica Bond RXm™ with the following modifications: After blocking in 10% (*v*/*v*) donkey serum, sections were incubated with rabbit anti-CD44 monoclonal antibody (1:100, Abcam #ab101531) and chicken anti-GFAP (1:1000, #b4674, Abcam) using antigen retrieval with Leica ER2 antigen retrieval buffer for 20 min. The secondary antibodies were Alexa Fluor^TM^ 488 donkey anti-chicken and 568 donkey anti-rabbit (1:500). Images were acquired at 40X (objective numerical aperture 0.65) on a Leica DMi8 Thunder system.

### 2.8. Quantification of CD44 in Hypoxia Sections

All CD44+ astrocytes were counted in 20X (numerical aperture 0.40) objective fields on a Nikon BX43 microscope. Three fields were counted per slide. Statistical analysis was performed using ANOVA. Data are shown as mean ± SEM. For quantification of the proportion of astrocytes that are CD44+ in different brain regions, we used QuPath to first quantify GFAP+ astrocytes using positive cell detection, then trained a random tree classifier to classify astrocytes as CD44+ or CD44−. The training data for the classifier included many examples of positive and negative cells derived from all of the slides examined.

### 2.9. Quantification of CD44 in Pilocarpine Sections

GFAP+ astrocytes were counted as either CD44+ or CD44− in the cortical area that contained double-labeled astrocytes. All GFAP+ astrocytes were counted in an area 221 × 221 μm^2^ with a 20X objective lens (objective numerical aperture 0.40) of the confocal microscope, and the percentage of all GFAP+ astrocytes that were also CD44+ was quantitated.

### 2.10. Statistics

We performed all statistics in GraphPad Prism 9 (GraphPad Software, Boston, MA, USA) or Miscrosoft Excel. We did not perform a power analysis because the experimental studies are well known. The post hoc statistical analysis examined a large number of astrocytes in several animals and showed a highly significant effect. Unpaired Student *t*-tests were used to compare test groups, hypoxia vs. control for the tMCAO study and pilocarpine vs. controls for the seizure study, as indicated in the text. For reporting the percentage of astrocytes that were CD44+, we did not perform statistical tests. All data are presented as mean ± standard error of the mean unless otherwise indicated.

## 3. Results

### 3.1. Cell Specificity of CD44 Immunostaining for Astrocytes

CD44 co-localized with GFAP in the isocortex and hippocampus [6]. Neurons, oligodendrocytes, myelin, and microglia were CD44-negative, although macrophages and classes of lymphocytes were positive. None of the autopsy brains we examined for the characterization of CD44+ astrocytes in non-pathological tissues had any neuropathological findings, including areas of macrophage accumulation, lymphocyte infiltration, or astrocyte scars.

### 3.2. Isocortex

**Figure 1:**
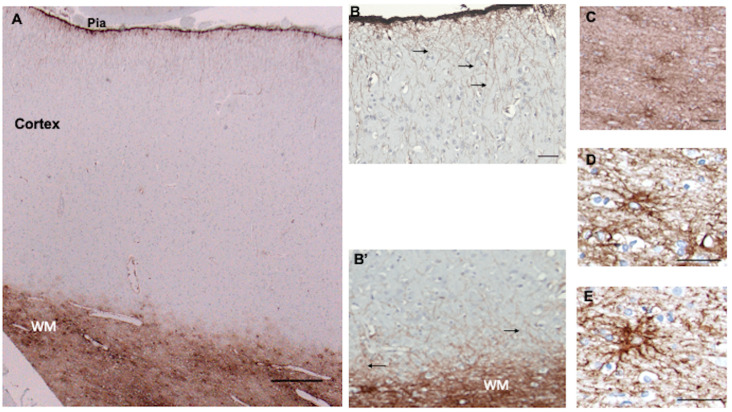
Superior frontal cortex shows CD44 positivity in subpial (Pia), interlaminar astrocytes, and white matter (WM) astrocytes, but not in the protoplasmic astrocytes of the cortex (**A**). Higher magnification of the pial surface and upper cortex shows the long interlaminar processes, radial to the pial surface (arrows) (**B**). Higher magnification of the white matter and lower cortex shows long astrocyte processes emerging into the cortex (arrows) (**B’**). (**B**,**B’**) are not from (**A**) but from other specimens, but all cortical specimens show the same pattern. The subcortical white matter contains dense astrocyte processes (**C**). Higher magnification of white matter astrocytes, which extend long processes in many directions, although the longest processes are roughly parallel and in the direction of axonal tracts (**D**,**E**). All specimens are counterstained with hematoxylin. Scale bars: (**A**) 250 μm, (**B**) 50 μm, (**C**) 25 μm, (**D**,**E**) 20 μm.

The cellular architecture of CD44+ astrocytes has been described in the isocortex [5,6,14] and matches that of the well-known interlaminar astrocytes with cell bodies at or near the pial surface and also astrocytes around large blood vessels. Note that the CD44+ astrocyte cell bodies sit at or near the pial surface (Figure 1). Isocortical neurons are not apposed or surrounded by CD44+ processes, including the Betz cells of the primary motor cortex (see below, in contrast with brainstem and spinal cord motor neurons, which are surrounded by CD44+ processes).

### 3.3. Subcortical White Matter

The fibrous astrocytes of white matter were CD44+ in every area of the CNS. These astrocytes were complex in shape, extending long processes along axonal tracts and shorter processes in other orientations as well (Figure 1C–E). As we have noted previously [6], some of the subcortical white matter astrocytes projected long, unbranched, radially oriented processes into the upper cortical levels (Figure 1B’). A number of other CNS areas also showed the projection of long CD44+ processes from white matter into gray matter (e.g., see dentate nucleus below).

### 3.4. Striatum

**Figure 2:**
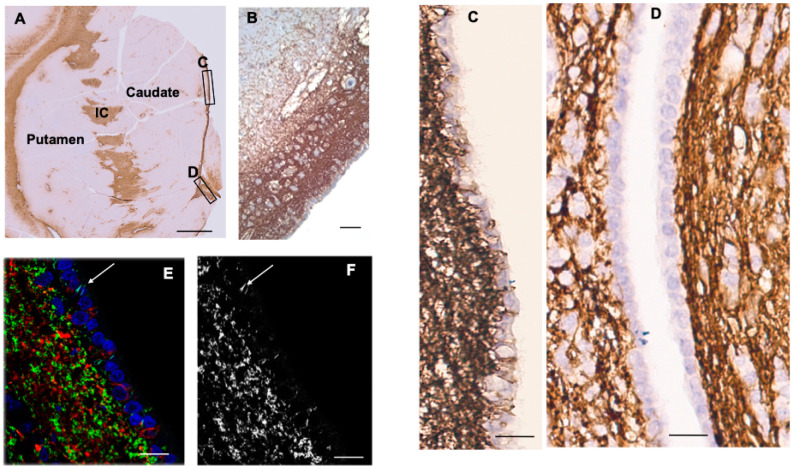
Caudate and putamen show CD44 positivity in the subependymal region, the internal capsule (IC), and the white matter fibers of the striatum (**A**). Higher magnification of the subependymal regions shows a dense network of CD44+ processes, some of which extend into the caudate (**B**). Higher magnification of the subependymal network (**C**,**D**). CD44+ processes extend between ependymal cells over the dorsal caudate to the ventricle in the dorsal area (**C**), but far fewer of these are present in more caudal areas (**D**). Confocal imaging also reveals CD44+ processes in between ependymal cells (arrows) (**E**, CD44—green, GFAP—red, DAPI—blue, (**F**, CD44+ only). Specimens (**A**–**D**) are counterstained with hematoxylin. Scale bars: (**A**) 5 mm, (**B**) 100 μm, (**C**–**F**) 20 μm.

CD44+ astrocytes occupied the subependymal layer, forming a dense matrix (Figure 2A–F). From there, long processes of CD44+ astrocytes were projected into the caudate nucleus. In examining the ependymal lining, we found that CD44+ processes intercalated between the CD44-negative ependymal cells (Figure 2B–F). Confocal imaging confirmed this finding (Figure 2E,F). In the ependymal lining over the caudate nucleus, these processes were more frequent dorsally than ventrally (Figure 2C,D). We observed this dorsal–ventral gradient in 11 of 11 sections of anterior caudate in patients ranging in age from 1 year to 94 years. In addition, we examined the germinal matrix at the angle of the lateral ventricle in eight fetal brains, aged 19 to 40 weeks of gestation, and four postnatal brains, aged 1 day to 7 weeks. All showed CD44+ processes in between ependymal cells (Supplemental Figure S1). In some cases, it was possible to trace these processes into the underlying subependymal zone. CD44+ astrocytes also appeared in the striatal white matter in both the pencil fiber bundles and the internal capsule, as expected. Protoplasmic astrocytes of the striatal gray matter were CD44-negative. No caudate or putamen neurons were surrounded by CD44+ processes.

### 3.5. Thalamus

**Figure 3:**
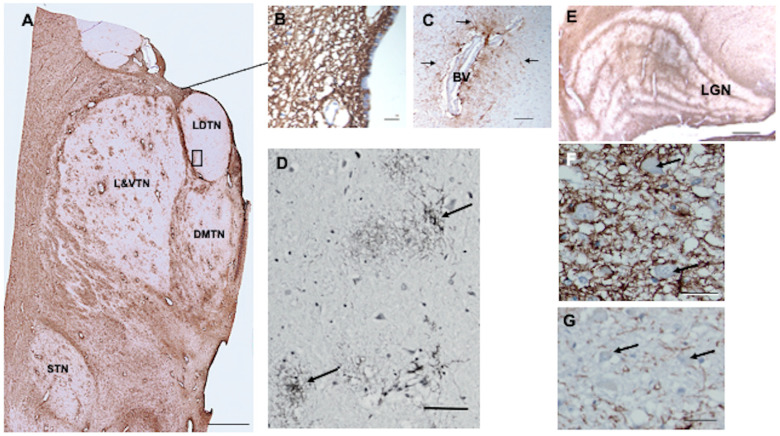
The anterior thalamus, including the latero-dorsal (LDTN), dorso-medial (DMTN), and latero-ventral (L&VTN) nuclei and the subthalamic nucleus (STN) show CD44 positivity around blood vessels and in white matter striae (**A**). CD44+ processes intercalate between ependymal cells (the line from A to B denotes the ependymal and subependymal area shown in (**B**)). Higher magnification of the boxed area in (**A**) shows a large blood vessel surrounded by CD44+ astrocytes, which send long unbranched processes into the parenchyma (arrows indicate some of the many processes) (**C**). Some of the CD44+ astrocytes in the L&VTN do not apparently contact blood vessels (arrows, (**D**)). Myelinated fiber bands of the lateral geniculate nucleus (LGN) are CD44+ (**E**). CD44+ surrounds magnocellular neurons (arrows, (**F**)) but not parvicellular neurons (arrows, (**G**)). All specimens are counterstained with hematoxylin. Scale bars: (**A**) 5 mm, (**B**) 10 μm, (**C**,**D**) 50 μm, (**F**,**G**) 20 μm.

In paraventricular thalamic nuclei, there was a dense network of CD44+ processes in the subependymal zone (Figure 3A,B). CD44+ processes also intercalated between ependymal cells (Figure 3B). Astrocytes surrounding large thalamic blood vessels appeared CD44+ and sent long processes into the thalamic neuropil (Figure 3C). There were more CD44+ astrocytes dispersed throughout thalamic nuclei than in the striatum. This may correlate with the large numbers of groups of myelinated axons that course through the thalamus, although some CD44+ astrocytes appear to be unassociated with white matter or blood vessels (Figure 3D). The anterior and lateral dorsal thalamic nuclei were relatively poor in CD44+ astrocytes, whereas the lateral nuclei and the subthalamic nucleus contained more of them (Figure 3A). Thalamic neurons were not surrounded by CD44+ processes, however. In the lateral geniculate nucleus (LGN), the white matter between layers contained CD44+ astrocytes, which extended long processes into the neuronal layers (Figure 3E). The large neurons of the magnocellular layers I and II were surrounded by CD44+ staining, while the small neurons in the other layers were not (Figure 3F,G).

### 3.6. Hypothalamus, Third Ventricle

As in other paraventricular areas, there was a dense subependymal network of CD44+ processes and an extension of long, unbranched processes from this into the parenchyma (Supplemental Figure S2A). As with the lateral ventricle, there were apparently more CD44+ processes intercalating between ependymal cells in the dorsal aspect compared with the ventral aspect of the third ventricle (Supplemental Figure S2B,C). Neurons of the paraventricular nuclei of the hypothalamus were completely or partially surrounded by CD44+ processes (Supplemental Figure S2D).

### 3.7. Hippocampus

**Figure 4:**
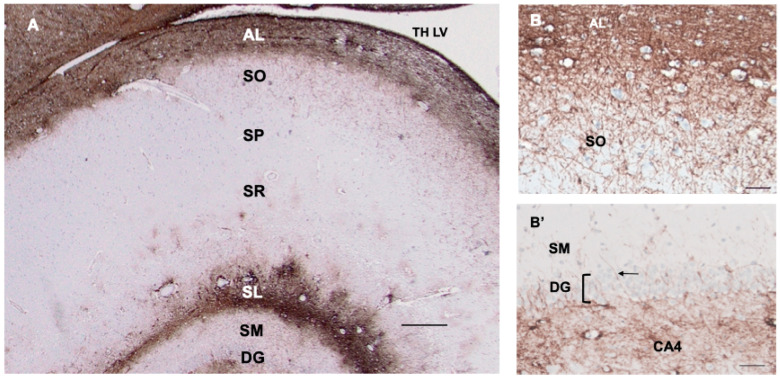
A section through the layers of the hippocampus (**A**) show CD44 positivity in the alveus (AL) and stratum lacunosum (SL). Thin, unbranched CD44+ processes run through the stratum oriens (SO) toward the pyramidal layer (stratum pyramidale, SP) (**A**,**B**). CD44+ processes do not surround pyramidal cells. Astrocytes in the stratum pyramidale, stratum radiatum (SR), and stratum moleculare (SM) show little CD44 reactivity. Dentate gyrus (DG). A higher magnification (**B**) shows the radially oriented processes in the SO. A higher magnification also shows the CD44+ processes (arrow) emanating from the subgranular zone of the CA4 sector through the dentate gyrus (DG, bracket) into the stratum moleculare (**B’**). (**B**,**B’**) are from a different specimen than (**A**), but all hippocampal specimens show the same patterns. Temporal horn of the lateral ventricle (TH LV). All specimens are counterstained with hematoxylin. Scale bars: (**A**) 250 μm, (**B**,**B’**) 200 μm.

As described [6], CD44+ astrocytes in the stratum oriens projected long processes through the depth of the pyramidal layer of CA1–CA3 and subiculum, ending at the border of the stratum pyramidale, which contains the cell bodies of the pyramidal neurons (Figure 4A,B). The pyramidal neurons were not surrounded by CD44+ processes. CD44+ astrocytes were localized to the molecular layer of the dentate gyrus, in which processes of astrocytes in the subgranular layer extended through the molecular layer (Figure 4B’). CD44+ astrocytes occupied the stratum lacunosum, the white matter tract from the entorhinal cortex. Note that both white matter tracts, the alveus (AL) and the stratum lacunosum (SL), are CD44+ labeled, as are all other white matter tracts in the CNS.

### 3.8. Cerebellum

**Figure 5:**
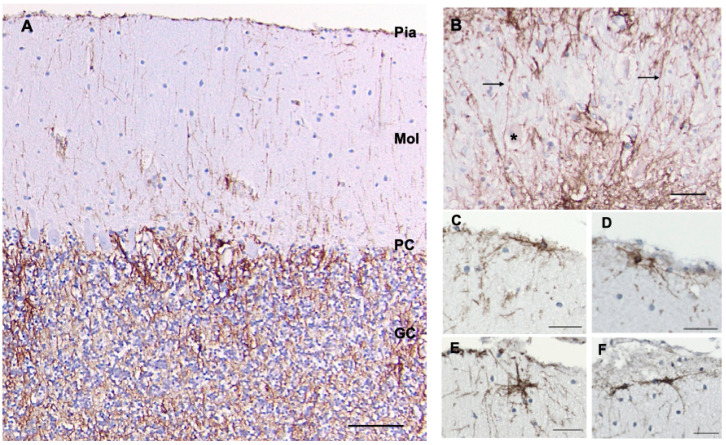
A section through the cerebellar cortex shows CD44+ long processes arising from the pial surface and entering the molecular layer (**A**). Some appear to begin in the granule cell layer and project into the molecular layer (see Supplemental Figure S3D). Fine CD44+ processes course through the granule cell layer, surrounding groups of granule cells, but do not surround individual granule cells. CD44+ processes (arrows) run through the dentate nucleus but do not surround dentate neurons (one labeled with *) (**B**). CD44+ astrocytes at or near the pial surface of the molecular layer extend processes parallel to the pia or into the molecular layer (**C**–**F**). Molecular layer (Mol), purkinje cell layer (PC), granule cell layer (GC). All specimens are counterstained with hematoxylin. Scale bars: (**A**) 100 μm, (**B**–**F**) 20 μm.

A. Cortex—We found cell bodies of CD44+ cells located either at the pial surface or just below it (Figure 5A,C–F). Many processes extended radially toward the Purkinje cell layer (Figure 5A and Supplemental Figure S3D), although we found that many cells also showed processes that were parallel to the pia (Figure 5C–F). The granule cell layer itself contained a network of CD44+ processes from the velate astrocytes, the astrocytes that populate this layer, and surrounding groups of granule cells, but not individual granule cells (Figure 5A and Supplemental Figure S3E,F). Finally, the spatial relationship of Purkinje cells to CD44+ processes seemed to vary. Some were not surrounded by CD44+ processes, although a few processes closely traveled by the lateral aspects of Purkinje cells, while others appeared to be surrounded (Supplemental Figure S3A–C). CD44+ processes extended radially from the granule cell layer into the molecular layer (Supplemental Figure S3D), indicating that some of the thin processes in the molecular layer had their origin in the granule cell layer. Moreover, in the Purkinje cell layer, a small subset of Bergmann glia, which sends long GFAP+ processes all the way to the pial surface, was CD44+ (Supplemental Figure S4D–F).

B. Dentate nucleus—The white matter surrounding the dentate nucleus, like other white matter in the CNS, was CD44+. Thin CD44+ processes entered and sometimes crossed the dentate nucleus and occasionally enwrapped some dentate neurons but not others (Figure 5B and Supplemental Figure S4A–C).

### 3.9. Brainstem

**Figure 6:**
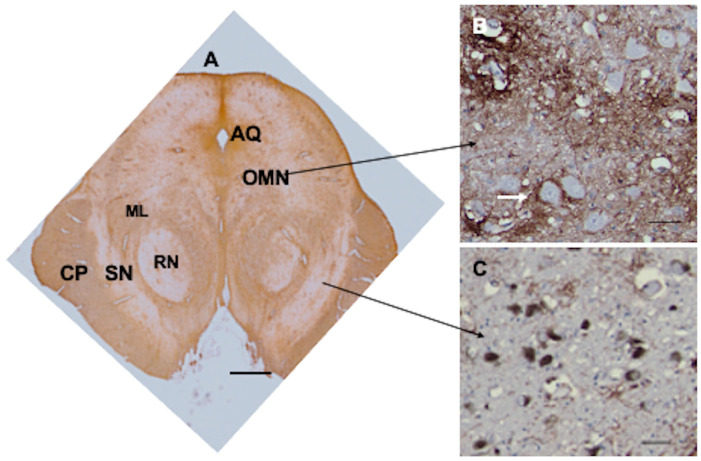
The midbrain appears complex, with CD44 staining of white matter tracts such as the cerebral peduncles (CP) and medial lemniscus (CP). The red nucleus (RN) contains CD44+ astrocytes. The substantia nigra (SN) appears relatively free of CD44+ staining (**A**). Neurons of the oculomotor nucleus (OMN) are surrounded by CD44+ staining (arrow shows one of these neurons) (**B**), but neurons of the substantia nigra are not (**C**). Aqueduct of Sylvius (AQ). Scale bars: (**A**) 5 mm, (**B**,**C**) 25 μm.

Many areas of the brainstem contained CD44+ astrocyte cell bodies and processes, in part due to the abundance of white matter pathways. In the midbrain, white matter pathways, such as the cerebral peduncles and the medial lemniscus, were all CD44+ (Figure 6). The red nucleus contained many CD44+ astrocytes, likely correlating with the myriad of myelinated axon bundles, but neurons were not surrounded by CD44+ processes. The cell bodies of motor neurons of the oculomotor nucleus were in close contact with CD44+ processes, in some cases enwrapped by them (Figure 6B). The substantia nigra contained few CD44+ astrocytes, and nigral neurons were not enwrapped by CD44+ processes (Figure 6C).

**Figure 7:**
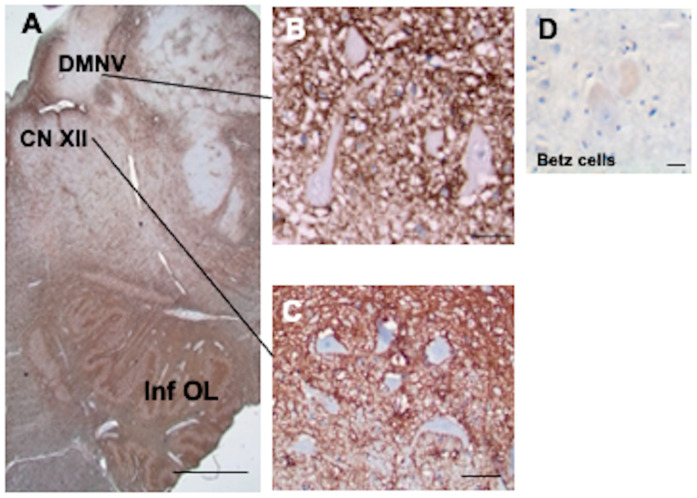
The medulla contains many CD44+ processes, including white matter tracts (**A**). Motor neurons of the dorsal motor nucleus of the vagus (**B**) and hypoglossal nucleus (**C**) are surrounded by CD44+ processes. In contrast, Betz cells of the motor cortex are not surrounded by CD44+ processes (**D**). Hypoglossal nucleus (CN XII), dorsal motor nucleus of the vagus (DMNV), inferior olivary nucleus (Inf OL). All specimens are counterstained with hematoxylin. Scale bars: (**A**), 2 mm, (**B**,**C**) 10 μm, (**D**) 50 μm.

The medulla also contained CD44+ astrocytes in white matter tracts (Figure 7A). Neurons of the hypoglossal nucleus (Figure 7B), the dorsal motor nucleus of the vagus (Figure 7C), the nucleus ambiguous, but not the motor neurons of the primary notor cortex (Betz ells) (Figure 7D) or the solitary tract nucleus or trigeminal nuclei, were surrounded by CD44+ processes (see Table 1 for a list of neurons that are and are not surrounded).

### 3.10. Spinal Cord

**Figure 8:**
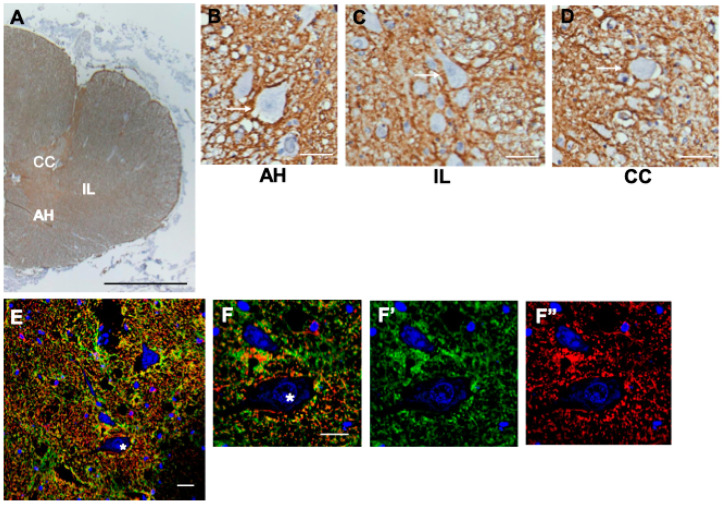
A hemi-section through the thoracic spinal cord, dorsal at top, ventral at bottom, shows many CD44+ processes, including white matter tracts (**A**). Motor neurons of the anterior horn (**B**), intermediolateralis (**C**), and Clarke’s column (**D**) are surrounded by CD44+ processes. One neuron in each panel is shown by an arrow. Anterior horn (AH), nucleus intermediolateralis (IL). Clarke’s column (CC). Specimens in (**A**–**D**) are counterstained with hematoxylin. Double immunolabeling for CD44—green and GFAP—red, showing low magnification of ventral horn (**E**, motor neuron *) and high magnification of the same anterior horn motor neuron (**F**, double stain, **F’**, CD44, **F’’**, GFAP). Scale bars: (**A**), 5 mm, (**B**–**F**) 20 μm.

The CD44 immunostaining of the spinal cord revealed CD44+ astrocytes in gray and white matter (Figure 8A–D). Motor neurons of the anterior horn were in close contact with CD44+ processes, in some cases enwrapped by them (Figure 8B). We found these contacts in all cord levels: cervical, thoracic, and lumbar. Neurons of the nucleus intermediolateralis (Figure 8C) and Clarke’s column (Figure 8D) were also surrounded by CD44+ processes. Double immunofluorescence labeling for CD44 and GFAP shows that CD44+ overlaps extensively with GFAP+, indicating that these processes belong to astrocytes (Figure 8 E,F). As a quantitative measure of neurons surrounded by CD44+ processes, we counted the numbers of anterior horn motor neurons in the CD44-immunostained spinal cords in eight separate autopsies (two anterior horns per section), each slide counterstained with hematoxylin to reveal cell nuclei and cytoplasm and found that all were surrounded by CD44+ processes (461/461).

**Figure 9:**
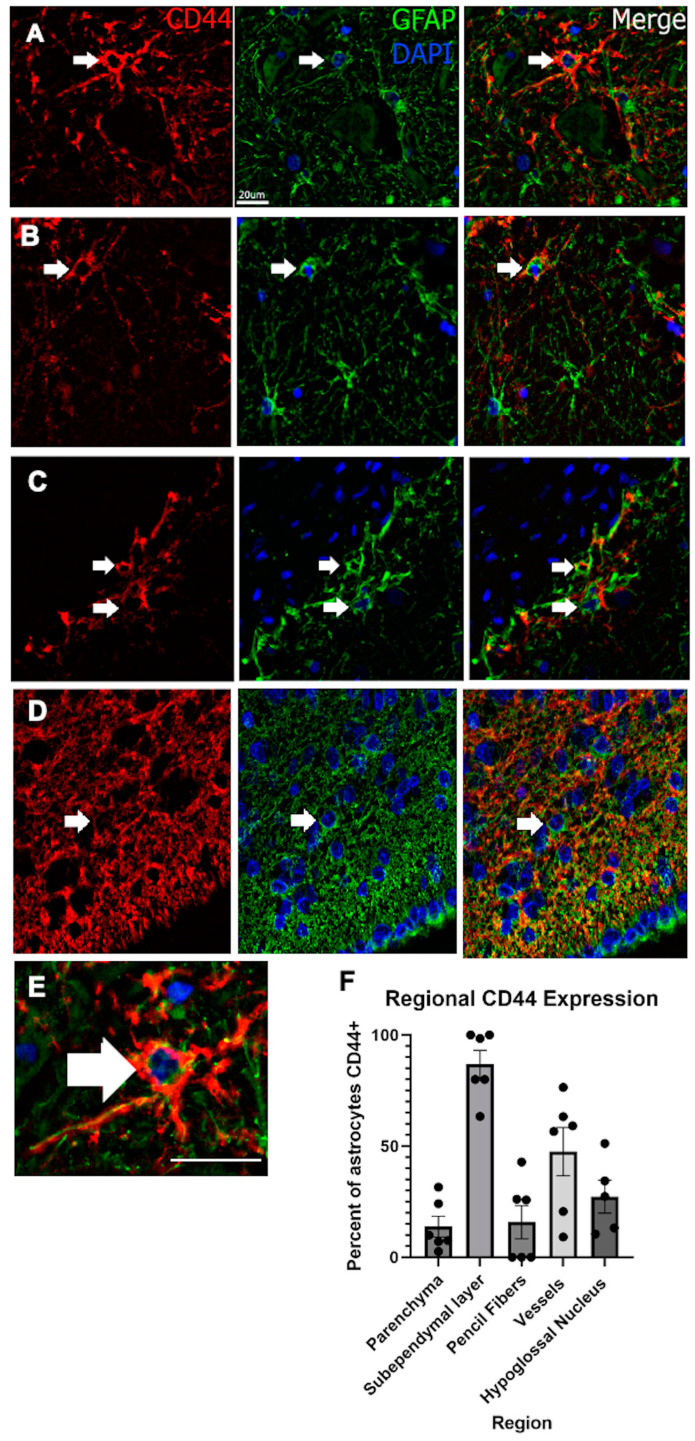
Examples of GFAP+/CD44+ double-labeled astrocytes in the hypoglossal nucleus (**A**), in the caudate parenchyma, excluding the pencil fibers (**B**), around small arteries (**C**), and the subependymal lining of the caudate nucleus (**D**). A higher magnification of A shows the double labeling (**E**). Note that the GFAP label localizes inside the CD44 label, the former being cytoplasmic and the latter membraneous. Note also that the CD44 extends beyond the GFAP since GFAP normally does not extend into the smaller distal processes of astrocytes. Quantitative measurements of double-labeled cells displayed as the percentage of GFAP+ cells that were also CD44+ (**F**). Arrows in all images show double-labeled cells. A total of 8045 astrocytes were counted in the parenchyma, 4482 in the subependymal layer, 21 in the pencil fibers, 670 next to vessels, and 1522 in the hypoglossal nucleus in a total of 6 different brains. Scale bar for panels (**A**–**E**) 20 μm (shown in (**A**) for the GFAP stain), but applicable for all images.

To quantitate the proportion of GFAP+ cells that are also CD44+, we counted the double-labeled cells in the caudate nucleus, including the parenchyma (gray matter only) subependymal region, the pencil fibers (white matter tracts), and around small arteries and then in the hypoglossal nucleus (Figure 9). Examples of double-labeled cells are provided in Figure 9A–E, and the quantitation of the proportion of GFAP+ cells that are also CD44+ in Figure 9F).

Finally, as a summary of all neurons that are or are not surrounded by CD44+ processes, we provide Table 1.

### 3.11. CD44+ Astrocytes in the Hypoxic Cortex

**Figure 10:**
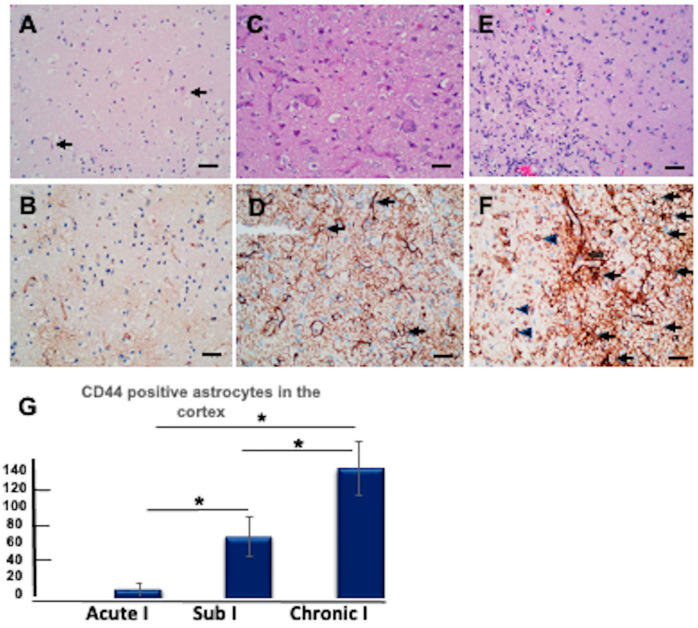
Protoplasmic astrocytes in the isocortex stain for CD44 following hypoxic/ischemic conditions. (**A**–**F**) represent acute, subacute, and chronic infarcts, respectively. Eosinophilic neurons (arrows) are seen in (**A**). Proliferative vasculature is present in (**C**). Foamy macrophages are more pronounced in (**E**) in an area of necrosis (left side of the panel). (**B**,**D**,**F**) are immunohistochemical stains for CD44 in the same tissue samples and hypoxic areas as shown in (**A**,**C**,**E**), respectively, but not the same fields. CD44+ astrocytes are highlighted (black arrows). Macrophages (arrowheads) are also positive for CD44. The vessels are outlined by CD44 stain. Specimens in (**A**,**C**,**E**) are stained with hematoxylin and eosin. Specimens in (**B**,**D**,**F**) are counterstained with hematoxylin. Scale bars: 20 μm. (**G**) The numbers of CD44+ astrocytes increase as infarcts evolve (average numbers in three 20X fields/specimen). Acute I: acute infarct; Sub I: subacute infarct; Chronic I: chronic infarct. (* *p* < 0.01, Sub I vs. Acute I, * *p* < 0.01, Chronic I vs. AI; * *p* < 0.01, Chronic I vs. Sub I). ANOVA with Tukey post hoc analysis. Values are shown as means ± SEM.

Protoplasmic astrocytes of the isocortex are not normally CD44+. To see if the protoplasmic astrocytes of the human cortex increase CD44 after hypoxia/ischemia, we examined by immunohistochemistry a series of neurosurgical and autopsy isocortical specimens from patients with well-defined clinical histories for the onset of hypoxic/ischemic events, allowing us to gauge the timing of CD44 accumulation (Figure 10). Very few CD44+ astrocytes were present in acute infarcts (1–2 days) (Figure 10A,B). However, within several days after the insult, some cortical astrocytes began to show weak staining for CD44 (Figure 10C,D), and by 12–14 days, many astrocytes in the affected cortex stained strongly (Figure 10E,F and Supplemental Figure S5). The CD44+ astrocytes resided in the penumbra of infarcts or within hypoxic lesions that did not contain necrosis.

**Figure 11:**
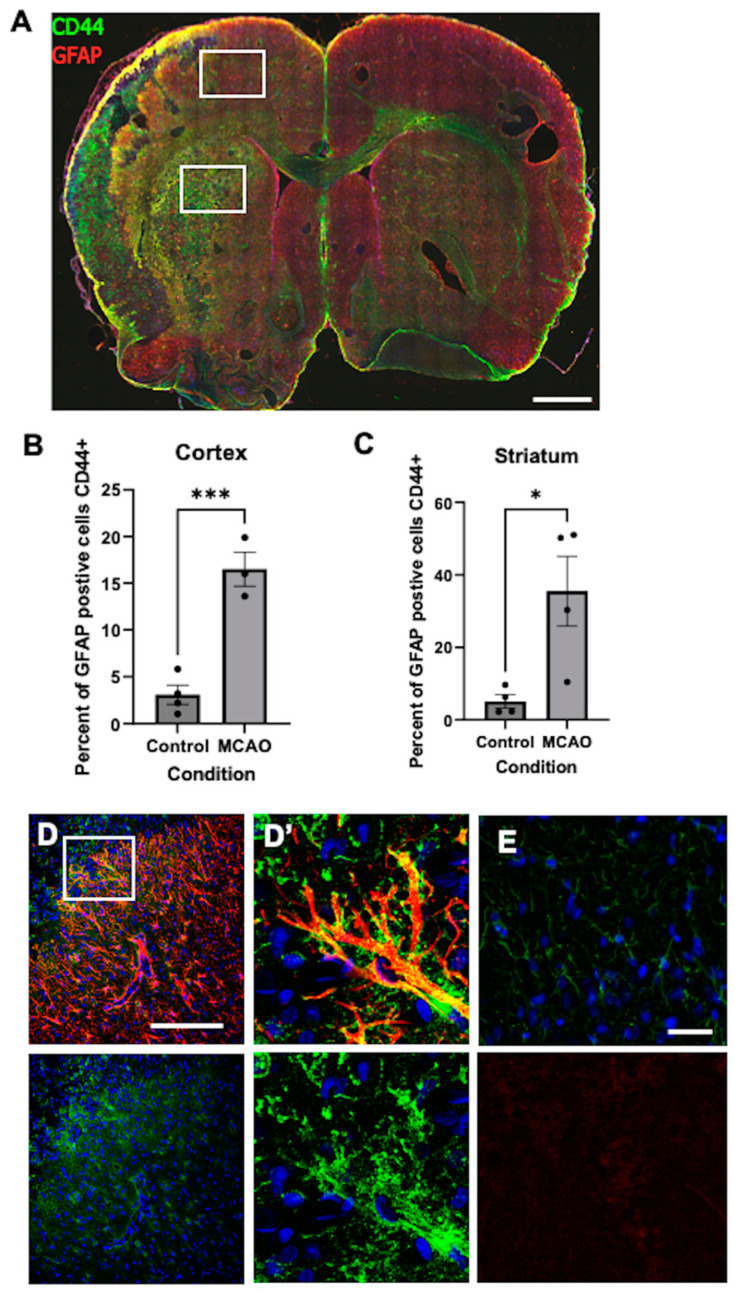
(**A**) Section of rat brain 7 days after left-sided tMCAO. Note the large number of CD44+-green signals on the left cortex and striatum and far fewer on the right side. This is a section from one of four separate experiments. The white rectangles delineate the areas in which GFAP+/CD44+ cells were counted for quantitation. Quantitative measurements of double-labeled cells are shown in (**B**,**C**), displayed as the percentages of GFAP+ cells that are also CD44+. * *p* = 0.021, *** *p* = 0.001, bars are SEM. Lower magnification (**D**) and higher magnification of boxed area (**D’**) show CD44+/GFAP+ astrocytes in the ipsilateral cortex. CD44—green, GFAP—red. The contralateral cortex only shows small numbers of lightly stained GFAP+ astrocytes; in this field, none is CD44+ (**E**). In (**E**), GFAP—green, CD44—red. In (**D**,**E**), the top row shows GFAP and CD44, and the bottom row shows only CD44 of the same field. A total of 6457 and 3004 astrocytes were counted in the hypoxic cortex and striatum, respectively, and 6212 and 1790 in the control cortex and striatum, in aggregate from 4 separate brains, each point is one brain. Scale bars: (**A**) 2 mm, (**D**) 240 μm, (**E**) 25 μm. The optically empty spaces in the right hemisphere in (**A**) are sectioning artifacts.

We also examined a rat model of brain ischemia generated by unilateral transient occlusion of the middle cerebral artery (tMCAO). After 7 days, many of the cortical and striatal astrocytes in the cortex and striatum ipsilateral to the MCAO, which had previously been GFAP and CD44-negative, became GFAP+, as expected after a hypoxic insult. Many of these also acquired CD44+. We show the hemispheres ipsilateral and contralateral to the tMCAO (Figure 11A, left vs. right, respectively) to illustrate the difference. The majority of GFAP+ cortical and striatal astrocytes on the contralateral side remained CD44-negative with the exception of the GFAP+ subpial astrocytes, which are normally CD44+, and some striatal astrocytes, as well as the astrocytes of the white matter. Quantitative counts of GFAP+/CD44+ cells show a significant difference (Figure 11B,C). Higher magnification of astrocytes illustrates details (Figure 11D,E).

### 3.12. CD44+ Astrocytes in Epilepsy

**Figure 12:**
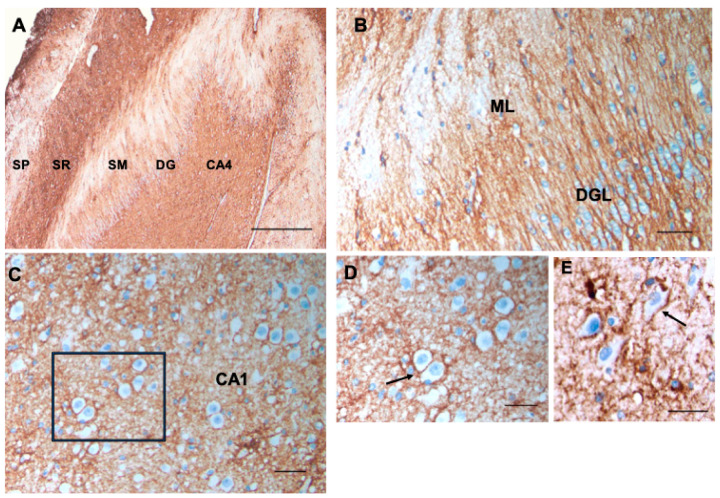
Changes in CD44 in the sclerotic hippocampus in human mesial temporal lobe epilepsy. Compare with Figure 4. There is an apparent increase in CD44 in all layers (abbreviations as in Figure 4) (**A**). The radially oriented astrocyte processes in the dentate granule layer (DGL), which extend into the molecular layer (SM), are CD44+ (**B**). Pyramidal neurons, which are normally not surrounded by CD44+ processes, have become so (**C**,**D**). (**D**) is the boxed area in (**C**). Pyramidal neurons are surrounded by CD44+ processes in another epilepsy specimen (**E**). Arrows represent pyramidal neurons surrounded by CD44+ processes. CA1, cornu ammonis 1, CA4, cornu ammonis 4. All specimens are counterstained with hematoxylin. Scale bars: (**A**) 200 μm, (**B**) 25 μm, (**C**) 25 μm, (**D**,**E**) 20 μm.

**Figure 13:**
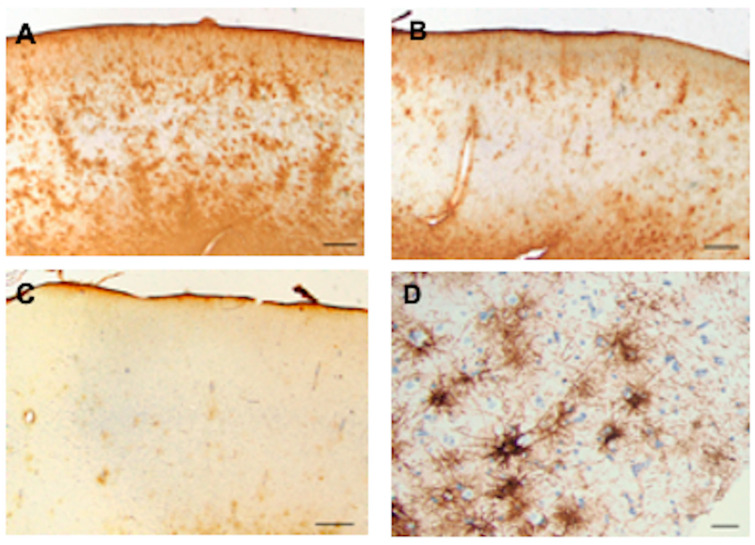
Temporal isocortex in individuals with temporal lobe epilepsy. In some resections, many astrocytes have become CD44+ (**A**). In others, fewer are CD44+ (**B**,**C**). The pial surface of the cortex is at the top. A higher magnification of CD44+ astrocytes in the temporal isocortex reveals that many of them have extended long processes, an abnormal morphology for protoplasmic astrocytes (**D**). The specimen in (**D**) is counterstained with hematoxylin. Scale bars: (**A**–**C**) 250 μm, (**D**) 20 μm.

Because of the reports of CD44 increase in epilepsy [15,16] and in tuberous sclerosis, which is often accompanied by seizures [14,17], we examined a group of neurosurgical specimens removed from patients with temporal lobe epilepsy, both the hippocampi and the adjacent temporal isocortex, for CD44+ astrocytes. The hippocampi showed an apparent increase in CD44 immunostaining in all sectors (Figure 12A,B) compared with controls (Figure 4). CD44 staining of all four resections for epileptic foci that contained the hippocampus revealed that neurons of the pyramidal cell layer were surrounded by CD44+ processes (Figure 12C–E), a morphology not present in the normal brain (Figure 4). The temporal isocortex showed a variety of intensities of CD44 staining in cortical astrocytes (Figure 13), although more appeared severe than moderate or low. We do not have the full clinical histories of all of these patients, however, so we cannot correlate the extent of CD44 staining with clinical behavior. Furthermore, sampling is an issue since we do not know how far away the cortical samples were taken from the hippocampus. Thus, we can only conclude that in some of these individuals, there has been a transformation of CD44-negative protoplasmic astrocytes to CD44+ astrocytes. Interestingly, some of the CD44+ cortical astrocytes had extended long processes (Figure 13D), an abnormal morphology for protoplasmic astrocytes, which normally do not extend such processes.

**Figure 14:**
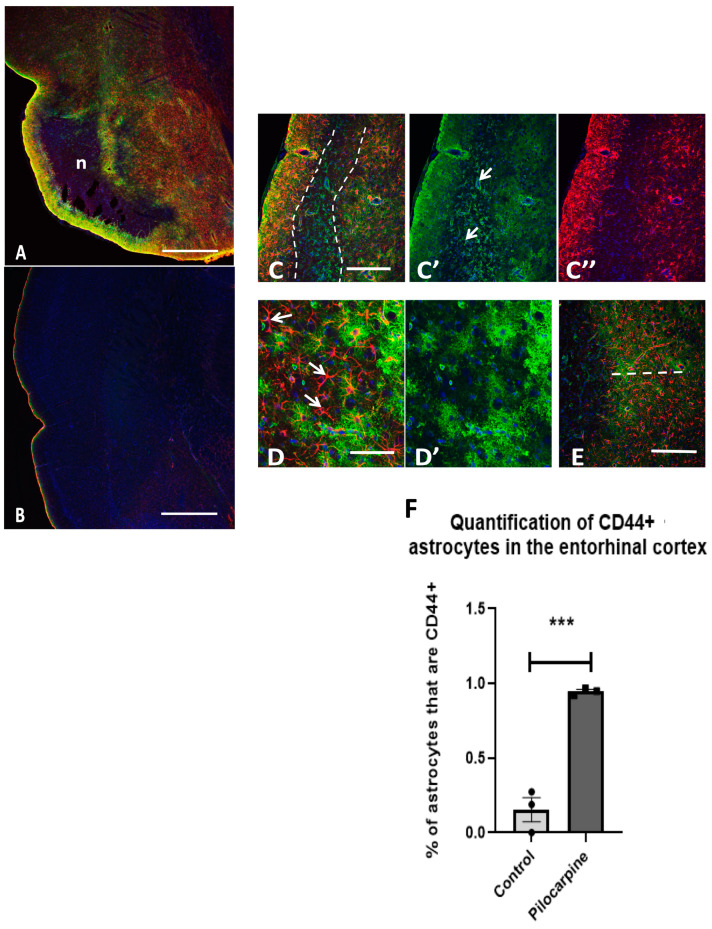
Cortical astrocytes become CD44+ in the rat entorhinal cortex 3 days after pilocarpine-induced seizures. A low magnification image shows strong GFAP+/CD44+ immunostaining in the subpial layer at the left of the image, no GFAP immunostaining in the necrotic layer (n) beneath the subpial layer, and strong GFAP+/CD44+ immunostaining in the layer beneath that (**A**). The normal entorhinal cortex in the control age-matched rat shows subpial astrocytes that are GFAP+/CD44+, but otherwise, there are very few double-labeled astrocytes (**B**). A higher magnification of the cortex shows the subpial gliosis, the necrotic layer outlined by the dotted line, and the gliotic layer beneath that (**C**). The necrotic zone contains CD44+ blood vessels (arrows) and round, CD44+ inflammatory cells (**C’**) but no astrocytes (**C’’**). The gliotic zone under the necrotic layer (right side of the image) contains astrocytes, many of which are GFAP+/CD44+ (**D**,**D’**). Arrows show GFAP+/CD44− astrocytes. Many are also double labeled, the CD44 showing the usually fine processes of the astrocyte domains. The width of the reactive astrocyte part of the cortex is denoted by a 500 μm dotted line in (**E**). In all images, GFAP—red, CD44—green, DAPI—blue. (**F**) Quantitative measurements of double-labeled cells displayed as the proportions of GFAP+ cells that were also CD44+. A total of 637 GFAP+ astrocytes were counted in 3 pilocarpine and 3 control brains. *** *p* = 0.001, bars are SEM. Scale bars: (**A**,**B**) 440 μm, (**C**) 220 μm, (**D**) 65 μm, (**E**) 220 μm.

We also examined the entorhinal cortex of rats that had been subjected to pilocarpine-induced seizures. The normal rat entorhinal cortex contains CD44+ astrocytes at the pial surface and in the subcortical white matter but not in the mid-cortex itself (Figure 14B). However, after the pilocarpine-induced seizures, a band of necrosis forms under the subpial area (delineated by “n” in Figure 14A and by dotted lines in Figure 14C). Note that there are no astrocytes in the necrotic zone. In the deeper cortical layers, there are many GFAP+/CD44+ astrocytes (Figure 14 C). The round CD44+ cells are inflammatory cells, and blood vessels in this area are also CD44+ (Figure 14C,C’). Higher magnification Figure 14D,D’) shows GFAP+ astrocytes, both CD44+ and CD44−. Note, as above, that the CD44 stain reveals the fine processes of astrocytes, not shown by GFAP since the filament does not extend into fine processes. Quantitative counts of GFAP/CD44+ astrocytes in the cortex show a highly significant difference between control and seizures (Figure 14F).

## 4. Discussion

### 4.1. CD44+ Astrocytes Populate the Entire Human CNS

In examining CD44+ astrocytes throughout the human CNS, we found not only that they populate every area but also that they are morphologically remarkably heterogeneous. Previous studies have reported CD44 immunostaining in the CNS, some of which localized it to astrocytes, but there has not been a systematic investigation of the entire human neuraxis in cellular detail [5,6,9,10,11,14,18]. Furthermore, we report novel observations of CD44+ processes intercalated between ependymal cells, subpial CD44+ astrocytes in the cerebellum, and CD44+ astrocyte processes that surround motor neurons and other large neurons.

### 4.2. Interlaminar Astrocytes

Long-process astrocytes in the isocortex have been known for over a century in human and other large mammalian brains. The smaller brains, such as those of rodents, do not show these, although astrocytes at the pial surface are also CD44+, as are white matter astrocytes. That the morphology is species dependent is implied by a study that produced long-process astrocytes in the mouse after transplanting human-induced pluripotent stem cells into the mouse cortex [19], suggesting that human long-process astrocytes are intrinsically programmed to assume this morphology.

Our findings in the isocortex and hippocampus confirm and extend those of our previous work [6]; we now show that CD44+ astrocytes are found throughout the human CNS. Long, unbranched processes lacking a bushy appearance distinguish the CD44+ astrocytes from the protoplasmic astrocytes of gray matter, which are CD44-negative. The presence of CD44, however, does not imply that all of these astrocytes represent a single population. For example, the “fibrous” astrocytes of the white matter may be different from the subpial, long-process astrocytes and the astrocytes that occupy subependymal locations or the astrocytes that wrap around brainstem and spinal cord neurons. Single-cell and single-nucleus RNA sequencing of the human CNS may indeed separate these populations, now defined by location and morphology, into multiple groups with different transcriptional profiles.

The pial-based CD44+ interlaminar astrocytes and the white matter-based astrocytes with processes entering the deeper cortical layers may represent remnants of the outer radial glia and ventricular radial glia that develop late in gestation in the human hemispheres after neuronal migration has taken place [20].

### 4.3. Neurons Surrounded by CD44+ Processes

One of the unexpected findings was that in the brainstem and spinal cord, many neurons are surrounded by CD44+ astrocyte processes. The presence of CD44+ processes surrounding motor neurons of the brainstem and spinal cord has not been previously reported. We did not see neurons surrounded by CD44+ processes in the isocortex, basal ganglia, thalamus, or hippocampus in the normal brain. Other large neurons that are surrounded by CD44+ astrocyte processes are the neurons of Clarke’s column, which are large, Nissl-rich neurons that receive input from large synapses from tendon organs and muscle spindles, and the magnocellular neurons of the LGN. Also, some Purkinje cells appear to be partially surrounded by CD44+ processes. Whether these contacts between neurons and CD44+ astrocytes are functionally important awaits further investigation. Studies of carcinoma cells may provide one clue to this interaction [21]. The intracellular domain of CD44 (CD44ICD) acts as a transcription factor for genes that contain a CD44ICD response element and promotes the expression of pyruvate dehydrogenase kinase (*PDK1*), 6-phosphofructo-2-kinase (*PFKFB4*), and aldolase C (*ALDOC*). Miletti-Gonzalez et al. [21] suggest that upregulation of these genes implies that CD44 may play a role in glycolysis since PDK1 promotes the conversion of pyruvate to lactate, a molecule known to be transferred from astrocytes to neurons to promote energy. Since these studies were performed in cancer cell lines, it would be interesting to test what genes the CD44ICD promotes in CD44+ astrocytes.

We found that hippocampal pyramidal neurons in temporal lobe epilepsy were also surrounded by CD44+ processes. We interpret this as the acquisition of CD44 by normal protoplasmic astrocytes whose processes normally contact neurons. Whether this represents a new process formation by astrocytes will await further investigation. What functional significance this enwrapping has will be interesting to examine.

### 4.4. Subependymal Astrocytes

Another unexpected finding was the dense matrix of CD44+ processes in the subependymal region. Because of the density, it was difficult to follow individual astrocyte morphologies, even with confocal microscopy. Future experiments injecting dyes into individual astrocytes would provide a better view of their morphologies. We did notice that CD44+ processes appeared to intercalate between ependymal cells, which themselves were CD44-negative. This morphology and location are reminiscent of neural stem cells in the adult mouse brain and subependymal astrocytes in the human brain, which can function as neural stem cells [22,23,24]. However, further comparison between these human CD44+ subependymal cells and neural stem cells will have to await further analysis.

### 4.5. Thalamic Astrocytes

Many of the CD44+ astrocytes in the thalamus and subthalamic nucleus are attached to large blood vessels, as seen elsewhere in the CNS. Other CD44+ astrocytes may be associated with myelinated fiber bundles not previously reported with CD44 immunostains.

### 4.6. Cerebellar Astrocytes

The cerebellar cortex contains several types of CD44+ astrocytes. Velate astrocytes, which have unique morphologies, send out many processes to enwrap clusters of granule cells [25]. Astrocytes in the upper part of the molecular layer, near the pial surface, are not well described to our knowledge. There are thin, radial, CD44+ processes passing through the molecular layer not previously described with CD44 immunostains. The source of these is not clear, although some are contiguous with CD44+ processes in the granule cell layer. Such thin processes were described by Santiago Ramón y Cajal, who thought, from his Weigert stains, that they originated from astrocytes in the granule cell layer or the underlying white matter [26]. These processes are far less dense than those of Bergmann glia, but some may represent a subpopulation of Bergmann astrocytes.

### 4.7. Astrocytes with Vascular Contacts

We also observed that CD44+ astrocytes are in contact with large blood vessels, sending long processes radially into the neuropil, as illustrated in Akiyama et al. [5]. In contrast, astrocytes that contact capillaries are CD44-negative. Thus, astrocyte contacts with arterioles may promote the CD44+ phenotype, or contact with capillaries may repress the phenotype and promote the characteristics of protoplasmic astrocytes. If CD44 is involved in the interaction, it may bind to the matrix surrounding large vessels, which is different from that around capillaries.

### 4.8. Hypoxia

We found that hypoxic/ischemic insults caused the protoplasmic astrocytes of the isocortex to become CD44+. Studies of tumor cell lines suggest mechanisms underlying this change. Thus, hypoxia regulates *CD44* expression via HIF-1α in breast cancer [27] and gastric cancer [28]. Furthermore, the CD44ICD is released in hypoxia by gamma secretase cleavage [29] and binds to HIF-2α [30]. This activates HIF-regulated genes. This latter study, which was performed with human glioblastoma cell lines, suggests a positive feedback mechanism in which hypoxia regulates *CD44,* which, in turn, may regulate more hypoxia-related genes. We found that cortical astrocytes in the rat brain become CD44+ after a tMCAO. In a previous study, *CD44* was found to be increased in the cortex and striatum of rats after a tMCAO, and localized macrophages and microglia [31], but the authors did not describe CD44+ in astrocytes.

### 4.9. Seizures

Seizures provoke an increase in CD44 in astrocytes, at least in the isocortex and hippocampus, the two areas we examined in the human CNS and in the mouse entorhinal cortex following pilocarpine-induced seizures. Furthermore, transcriptomics of the reactive astrocytosis accompanying temporal lobe epilepsy show upregulated *CD44* [16]. The mechanisms underlying this are not known. Hypoxia may be involved, or inflammatory responses. A previous study found CD44 in the mouse hippocampus after pilocarpine-induced seizures, but astrocyte localization was not described [15].

### 4.10. Regulation of CD44 That Might Be Relevant to Studies in the Human CNS

Although the transcriptional regulation of *CD44* has not been studied in human astrocytes, studies with other cultured cell types show that the upstream promoter region of CD44 is complex, containing binding sites for both positive and negative transcriptional regulators, including sequences that bind AP-1, NF-kB, Sp1, Egr1, TCF4, ETS-1, p53, KLF4, and Foxp3, the latter three as repressors [32,33]. As noted above, hypoxia regulates *CD44* through HIF-1α. These studies were carried out in non-CNS cancer cell lines, and it is not known whether similar regulations occur in astrocytes. Mouse astrocytes in culture upregulate *CD44* when treated with phorbol-12-myristate 13-acetate or TNFα plus gamma interferon [34], suggesting that inflammatory conditions may upregulate *CD44*. Which of these transcription factors acts to regulate *CD44* in human astrocytes is unknown.

In addition, there is evidence for epigenetic regulation of *CD44*. Thus, a 5′-CpG island of *CD44* is methylated in prostate cancer [35]. CpGs are highly methylated in the CD44-negative T cell lymphoma line AKR1, although transfection with *c-jun* can activate transcription without major changes in methylation [36]. It will be of interest to examine the methylation status of *CD44* in CD44+ and CD44-negative astrocytes and investigate if these changes in hypoxia or seizures allow *CD44* transcription.

### 4.11. There Is a Negative Correlation between CD44 and Protoplasmic Astrocyte Genes/Proteins

We previously determined that the CD44+ interlaminar astrocytes in the normal human isocortex have lower levels of glutamate transporters and glutamine synthetase than CD44-negative protoplasmic astrocytes [6]. In a mouse model of Alexander disease, a CNS degenerative disorder caused by mutations in *GFAP*, we found that protoplasmic astrocytes of the hippocampus and isocortex became CD44+ as the disease progressed [37]. The acquisition of CD44 correlated strongly with the loss of the plasmalemmal glutamate transporter, Glt-1. In a study of the human cingulate gyrus in Huntington disease, RNA sequencing revealed an upregulation of *CD44* during the disease [38]. This study also revealed that astrocytes lost the expression of normal protoplasmic astrocyte genes. Thus, there appears to be a negative correlation between CD44 and several protoplasmic astrocyte genes and proteins. Whether this correlation is caused by the presence of CD44 is not known, although it is testable in future studies. The expression of *CD44* caused by hypoxia and seizures may well alter the physiology of protoplasmic astrocytes in ways that are deleterious to neurons.

### 4.12. Is There Any Value in Downregulating CD44 in Pathological Situations?

If CD44 plays a role in changing the phenotypes of protoplasmic astrocytes, would there be any value in downregulating *CD44* or preventing its upregulation? Would this preserve a normal protoplasmic astrocyte state? This could be tested in mouse models. In studies of human cancers, CD44 is associated with STAT3, which may lead to STAT3 activation [39]. STAT3, in turn, activates other astrocyte genes, such as *GFAP* [40,41]. So et al. [39] found that a vitamin D analog represses *CD44* expression in breast cancer cells. It would be interesting to determine if astrocytes show the same response.

### 4.13. Limitations

Although this is the most thorough study of CD44+ astrocytes in the human CNS, and we show the conversion of protoplasmic astrocytes to being CD44+ in hypoxia and epilepsy, our observations do not imply specific molecular mechanisms. Such data on the mechanisms of *CD44* expression appear largely in the cancer biology literature and have not been investigated in astrocytes. The present study provides an important background that will promote further investigations by our labs and others.

## 5. Conclusions

This study includes a number of novel observations, further characterizing the heterogeneity of astrocytes in the human CNS.

1. CD44+ astrocytes are present at all levels of the human CNS.
2. Some neurons are surrounded by CD44+ processes, including motor neurons in the brain stem and spinal cord, while isocortical, hippocampal, and most diencephalic neurons are not.
3. CD44+ processes intercalate between ependymal cells at ventricular surfaces.
4. CD44 immunostaining revealed astrocytes in the upper part of the cerebellar molecular layer. These send processes parallel to the pia and radially toward the Purkinje cell layer.
5. Velate astrocytes of the cerebellar granule cell layer are CD44+.
6. Hypoxia and seizures induce CD44-negative protoplasmic astrocytes to become CD44+.

## Figures and Tables

**Table 1:** A list of neurons that are or are not surrounded by CD44+ processes.

Not Surrounded by CD44+	Surrounded by CD44+
Isocortex, including Betz cells	Oculomotor nucleus
Thalamus	Facial nucleus
Basal ganglia	Hypoglossal nucleus
Subthalamic nucleus	Dorsal motor nucleus of vagus
Hippocampal dentate gyrus	Lateral vestibular nucleus
Hippocampal and para-hippocampal pyramidal neurons	Inferior olivary nucleus
Locus ceruleus	Spinal anterior motor neurons
Red nucleus	Onufrowitz nucleus
Substantia nigra	Clarke’s column
Colliculi	Intermediolateralis nucleus
Pontine base	
Cerebellar granule cells	
Dentate nucleus +/−	
Purkinje cells +/−	
Solitary nucleus	

## Data Availability

There are no research data or data files other than the text and images presented in this paper.

## Supplementary Materials

The following supporting information can be downloaded at: https://www.mdpi.com/article/10.3390/cells13020129/s1, Figure S1: Sections through the ependymal lining overlying the subependymal zone in fetal brains to show CD44+ processes between ependymal cells, Figure S2: A coronal section through the lateral and third ventricles shows CD44+ in the anterior commissure (AC), optic tracts (OT), and internal capsule (IC), Figure S3: Sections through the cerebellar cortex, Figure S4: CD44+-positive astrocytes in the dentate nucleus (left panels, A–C) and cerebellar cortex (right panels, D–F), Figure S5: In an individual who died with acute hypoxia, isocortical neurons are eosinophilic and shrunken (arrows, A), but astrocytes are not CD44+ (B). In individuals with chronic hypoxia, cortical astrocytes become CD44+ (C–E).
