# Supplementary material for: The Matrix Receptor CD44 Is Present in Astrocytes throughout the Human Central Nervous System and Accumulates in Hypoxia and Seizures
Source: Cells. 2024 Jan 10;13(2):129. doi: 10.3390/cells13020129 (PMC10814649; doi:10.3390/cells13020129)
Supplement: Supplementary file 1 [file cells-13-00129-s001.zip › cells-2320300-SI.pdf]

Supplemental Materials:

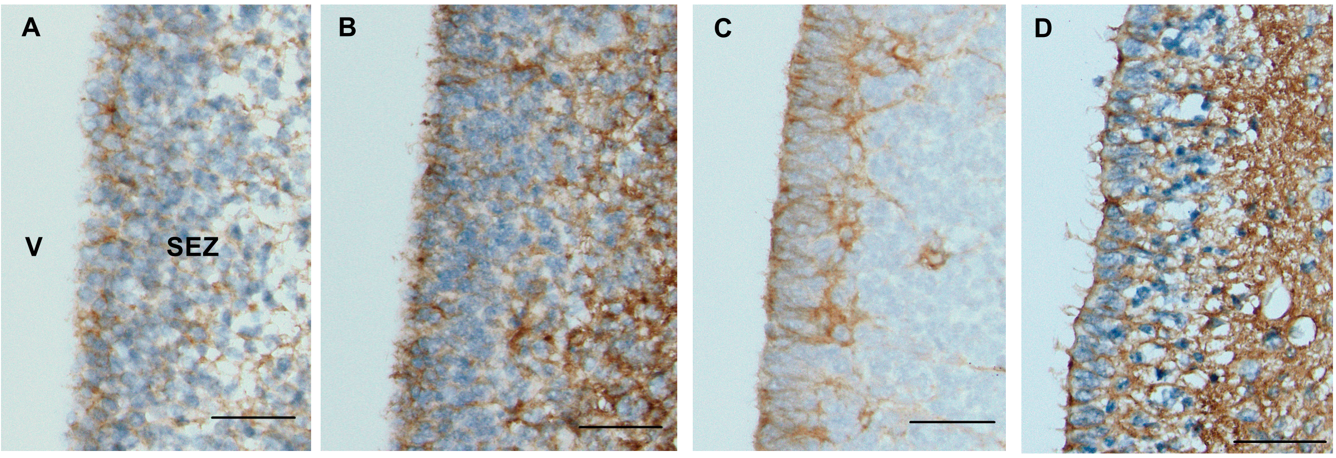

Supplemental Figure S1: Sections through the ependymal lining overlying the subependymal zone in fetal brains to show CD44+ processes between ependymal cells. Gestational ages – 19 weeks (A), 20 weeks (B), 33 weeks (C), 40 weeks (D). Lateral ventricle (V), subependymal zone (SEZ). Scale bars: A, B, C, D 20 µm.

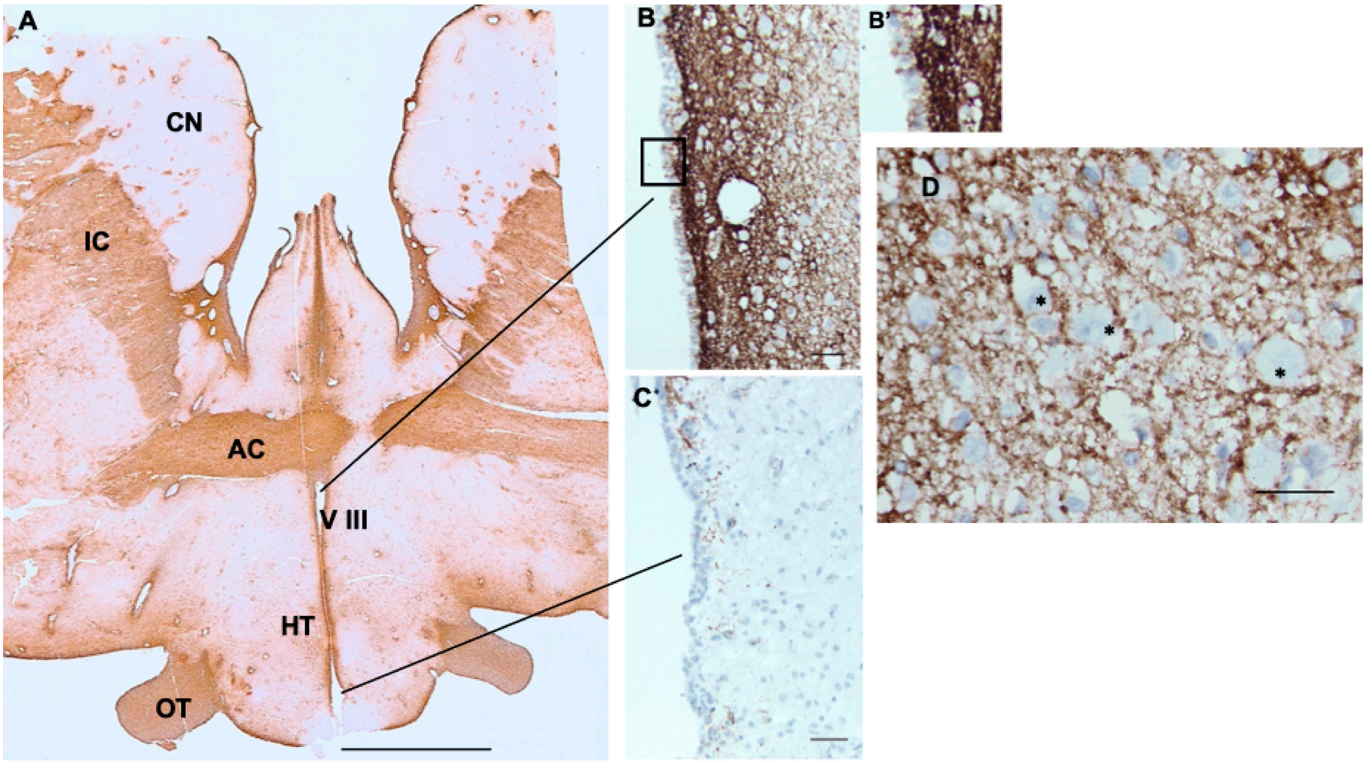

Supplemental Figure S2: A coronal section through the lateral and third ventricles shows CD44+ in the anterior commissure (AC), optic tracts (OT), and internal capsule (IC). (A). CD44+ processes extend between ependymal cells in the third ventricle (V III), more in the rostral (B) than in the caudal (C) region.

Inset (B′) shows the CD44+ processes between ependyma in the area outlined by the box (B) at higher magnification. Neurons of the paraventricular nucleus of the hypothalamus (HT) (some labeled *) are surrounded by CD44+ processes (D). Scale bars: A, 5mm, B, C 25 µm, D 20 µm.

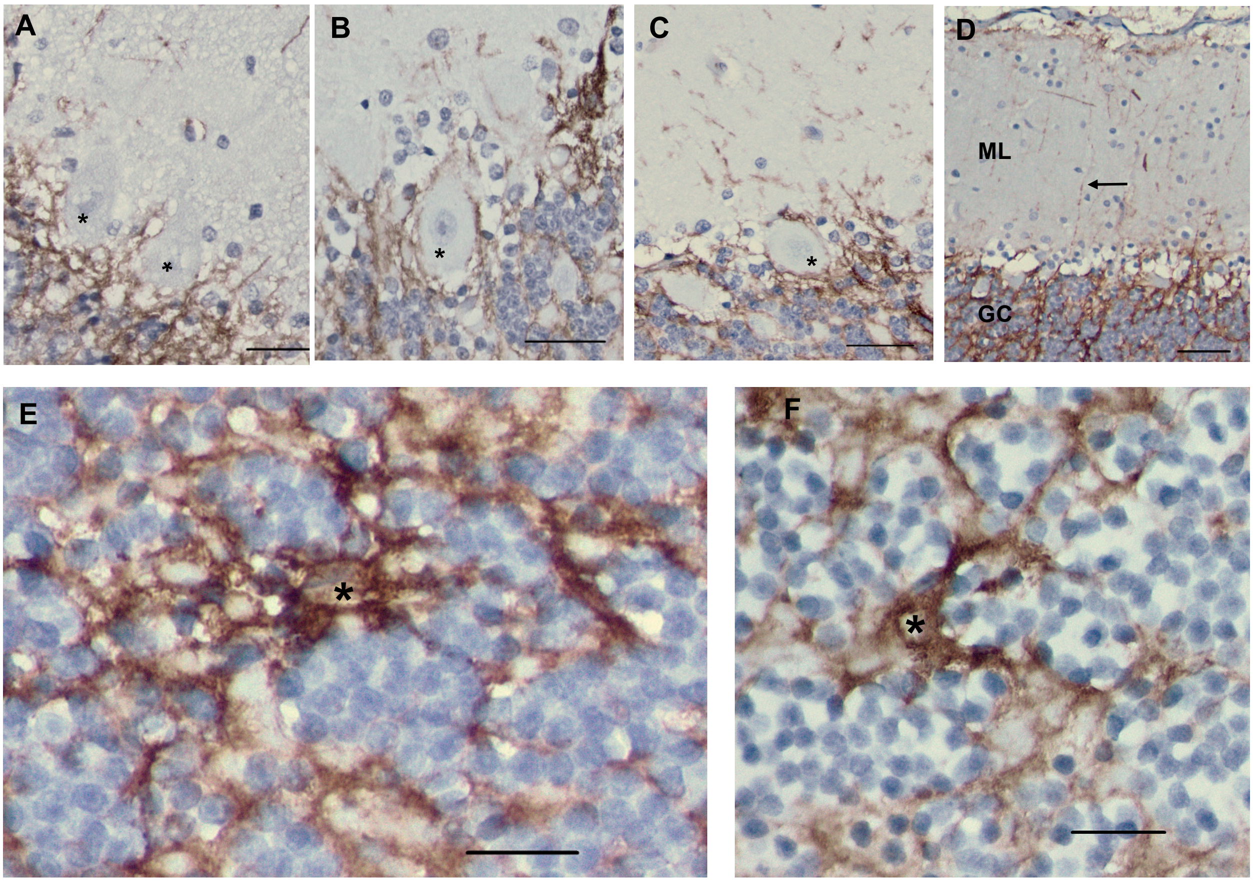

Supplemental Figure S3: Sections through the cerebellar cortex. Purkinje cells (*) are variably covered by CD44+ processes (A-C). Thin processes in the molecular layer (ML, arrow) are contiguous with CD44+ processes in the granule cell layer (GC) (D). (E, F) each shows one CD44+ velate astrocyte (*) in the granule cell layer. Note how their many processes surround nests of granule cells and not individual neurons. Scale bars: A-C 20 µm, D 25 µm, E 20 µm, F 10 µm.

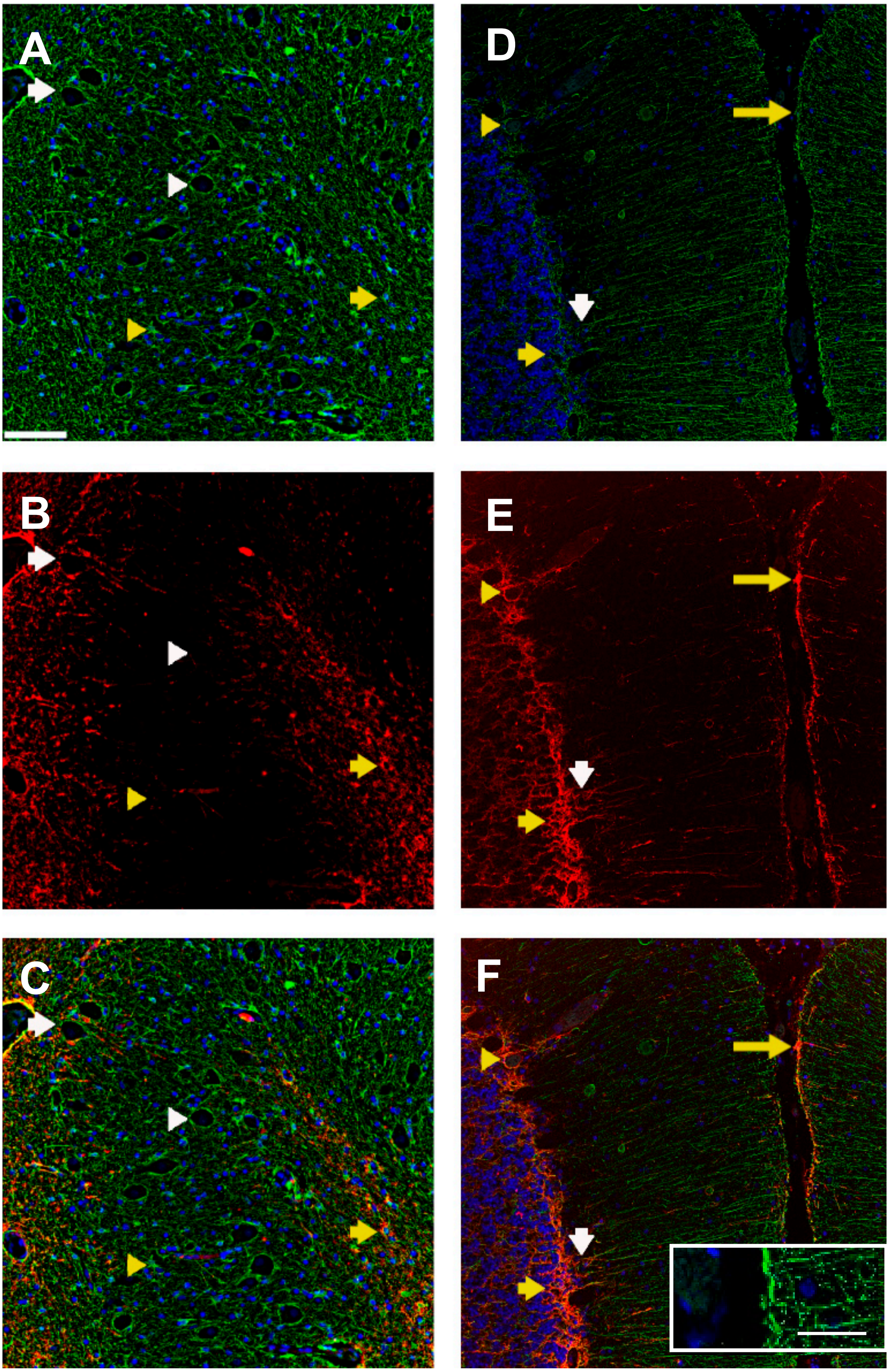
A
B
C
D
E
F

Supplemental Figure S4: CD44+ positive astrocytes in the Dentate Nucleus (left panels, A, B, C) and Cerebellar Cortex (right panels, D, E, F). (A, D) show DAPI (blue, nuclei) and GFAP (green). (B, E) show only CD44 (red). (C, F) is a mix of the three colors. In the Dentate Nucleus the white arrow indicates a neuron surrounded by CD44+, the white arrowhead indicates a neuron with surrounding GFAP but not CD44. The short yellow arrow shows a CD44+ astrocyte in the white matter and while the yellow arrowhead shows an astrocyte that is GFAP+ (C) but not CD44+ (B). In the cerebellar cortex, the arrowhead points to a Purkinje cell surrounded by CD44. The long arrow shows a CD44+ astrocyte at the pial surface with a process extending into the molecular layer (seen at higher magnification in the inset in F), the short yellow arrow points to CD44+ velate astrocytes with the white arrow indicating a CD44+ Bergmann astrocyte. Scale bar (in A) for all panels is 100 µm, Inset for F 50 µm.

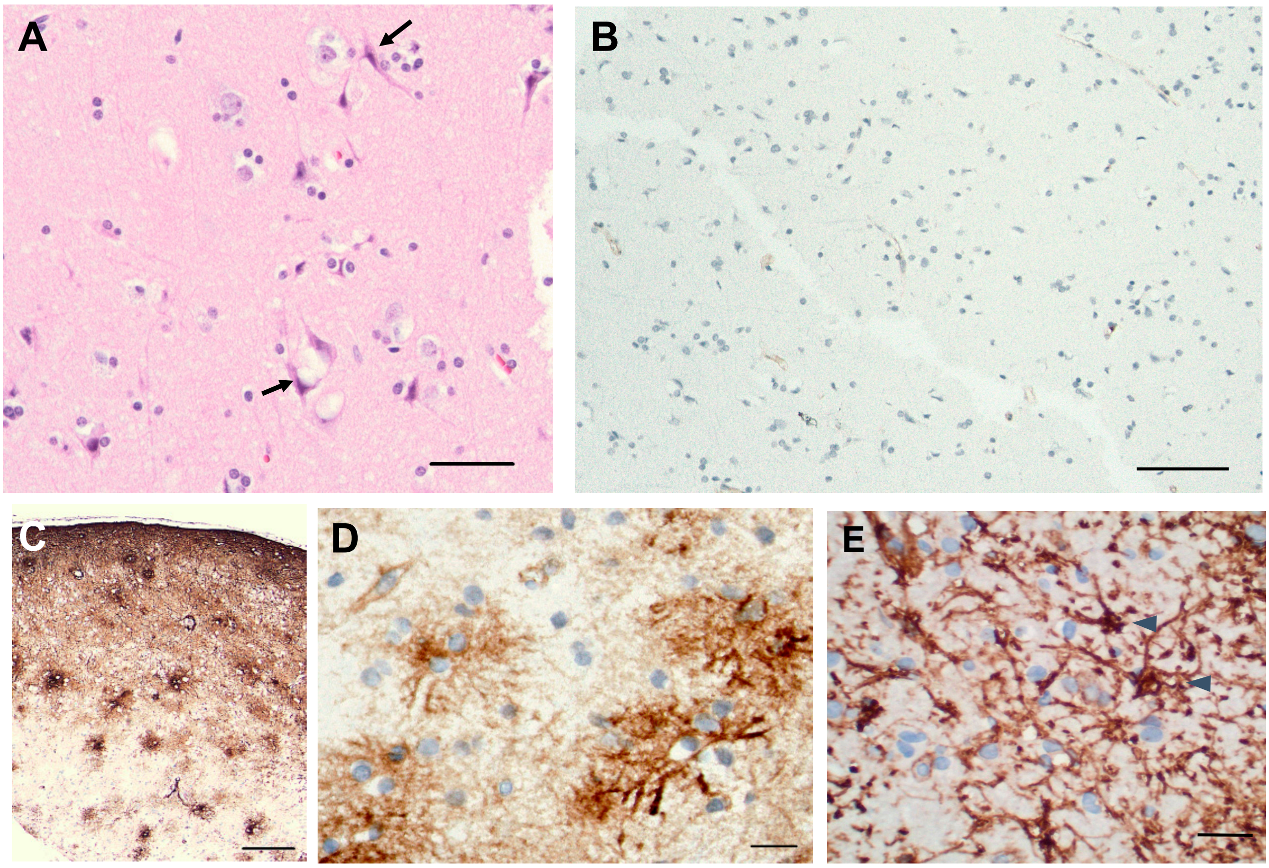

Supplemental Figure S5: In an individual who died with acute hypoxia, isocortical neurons are eosinophilic and shrunken (arrows, A), but astrocytes are not CD44+ (B). In an individual whose brain revealed chronic hypoxia in the cortex, many astrocytes are CD44+ (C), seen at higher magnification in (D and E). Scale bars: A 25 µm, B 50 µm, C 100 µm, D, E 10 µm.
